# From hands to minds: Gestures promote understanding

**DOI:** 10.1186/s41235-016-0004-9

**Published:** 2016-09-22

**Authors:** Seokmin Kang, Barbara Tversky

**Affiliations:** 1 Wisconsin Center for Education Research, University of Wisconsin–Madison, Educational Sciences Building, 1025 West Johnson Street, Madison, WI 53706 USA; 2 Department of Psychology, Stanford University, Stanford, CA, USA; 3 Department of Human Development, Teachers College, Columbia University, New York, NY, USA

**Keywords:** Congruent gesture, Explanation, Causal reasoning, Representation, Embodiment, Learning, Dynamic systems

## Abstract

Gestures serve many roles in communication, learning and understanding both for those who view them and those who create them. Gestures are especially effective when they bear resemblance to the thought they represent, an advantage they have over words. Here, we examine the role of conceptually congruent gestures in deepening understanding of dynamic systems. Understanding the structure of dynamic systems is relatively easy, but understanding the actions of dynamic systems can be challenging. We found that seeing gestures representing actions enhanced understanding of the dynamics of a complex system as revealed in invented language, gestures and visual explanations. Gestures can map many meanings more directly than language, representing many concepts congruently. Designing and using gestures congruent with meaning can augment comprehension and learning.

## Significance

Effective communication, notably teaching, is a central application of cognitive psychology. Explaining processes that occur over time is especially challenging, primarily because of the complexity of the sequence of actions and their causes and consequences. Adding gestures that are crafted to congruently represent the actions to the verbal explanation deepens understanding of the actions and the system as a whole. Gestures are especially effective because they can both resemble and represent and also embody action.

## Background

### Understanding dynamic systems

Mastering dynamic systems is a recurrent task in our lives. In school, learning the behavior of neurons, the growth of plants, the behavior of molecules, and the events leading to the French Revolution; in our everyday lives, filing income taxes, operating the proverbial VCR, and using new software; in our public lives, understanding the workings of the electoral college, the behavior of the stock market, and the actions of the various political and religious factions in the Middle East. These systems can be decomposed into parts, the actions of the parts over time, and the consequences of the actions; hence, the term “dynamic systems.” Grasping some dynamic systems is difficult because the systems are not thoroughly understood or probabilistic, but even well-understood dynamic systems are challenging. Dynamic systems ordinarily have one or more structural layers and one or more layers of action. Structural layers consist of a set of parts, typically with specific associated properties, and their interrelations. Layers of action, behavior, process or causality consist of sequences of kinds of actions and their consequences. The structural layer is static, and if only for that reason, is easier to understand. The action layer is dynamic; it consists of changes in time, specifically, a sequence of varying actions and outcomes that are the consequences of the actions, often accompanied by causal reasons. Smart undergraduates who happen to score below the median in a test of mechanical ability—that is half the undergraduates—have difficulties understanding the behavior of dynamic systems, even relatively simple ones like the workings of a car brake or bicycle pump or pulley system, though they readily grasp the structure of the system parts (e.g., Hmelo-Silver & Pfeffer, 2004; Tversky, Heiser, & Morrison, 2013). Understanding the behavior of dynamic systems entails comprehending the temporal sequence of the actions of the parts of the system, the nature of the actions, the changes that result, and the causal dependencies between the actions and the changes.

### Representing dynamic systems in graphics

The structural levels of dynamic systems, a configuration of parts, can be readily mapped to diagrams and that is, in fact, a common approach to representing them. Putting concepts into the world in the form of sketches, models, diagrams, artifacts and the like is well-known to promote memory, thinking and learning (e.g., Card, Mackinlay & Shneiderman, 1999; Hegarty, 2011; Larkin & Simon, 1987; Mayer, 2005; Schon, 1983; Tufte, 1983; Tversky, 2001, 2011). For simplicity, let us call the various forms of externalizing thought *graphics*. Putting and arranging thought in the world using graphics can spatialize that information as well as expand memory and promote information processing. Importantly, the ways that elements are shown and spatially arranged can abstract and structure thought more directly and congruently than language. The parts of a system that are close or interacting can be shown as close and interacting. The parts and whole can be depicted, as can some kinds of actions. Sequences of actions can be indicated by arrows. Representing the objects and arrangements of thought in the world provides a platform for inference and discovery (e.g., Tversky, 2011).

### Representing change over time in graphics

Graphics are for the most part static; they can stay in front of the eyes to be contemplated. Yet, exactly because graphics are static, conveying dynamic systems that entail action, process, behavior, or change in time, has proved challenging for graphics.

Several solutions have been devised to convey dynamic information in graphics, including arrows, successive still diagrams and animated diagrams; none have proved to be universally satisfactory. As noted, a common and often successful solution is to use arrows. People readily produce and interpret arrows as temporal and/or causal relations (e.g., Heiser & Tversky, 2006). However, arrows can be ambiguous because they have a multitude of uses in diagrams. They can be used to label, to indicate temporal sequence, to indicate movement, to indicate causal connection, to show invisible forces, and more (e.g., Tversky, 2011). Many diagrams in the social sciences, biological and physical sciences, and engineering use arrows in multiple ways without disambiguating their meanings, resulting in diagrams that can be confusing and difficult to comprehend (Tversky, Heiser, MacKenzie, Lozano, & Morrison, 2007). In addition, showing the qualitative properties of important kinds of actions, such as forming alliances or chemical bonding or explosions or condensation, takes more than arrows. Another common method to show change in time is a sequence of still diagrams; however, successive stills also have limitations. Like arrows, they cannot readily show qualitative aspects of actions. In addition, they require integrating the separate still diagrams in turn, not an easy task. The separate diagrams must be compared by eye, and the changes between them imagined. Yet another way to convey action is by animations. Animations are especially compelling because they are conceptually congruent with what they convey: they use change in time to convey change in time (Tversky, Morrison, & Betrancourt, 2002). However, a broad survey comparing animated and still graphics relaying the same information and designed to educate viewers about complex processes that occur over time showed no benefits from animated graphics (Tversky et al., 2002). Three reasons were proposed for the failure to find benefits of animated over static graphics for conveying processes in time. One reason for the lack of success of animated educational graphics is perceptual, too much happens at the same time, so it is hard to grasp the sequence and nature of the changes. Another shortcoming of most educational animations is that they do not break the changes into their natural units. Instead, they show change in time continuously, proportionate to real time. The explanations that teachers and lay people in general provide are not continuous in time and proportionate to real time. Instead, explanations provided by people generally break processes into natural steps. Here is a simple example: when explaining routes, people segment them as a sequence of turns at landmarks (Denis, 1997; Tversky & Lee, 1998). Similarly, in describing actions that are continuous in time, like doing the dishes or making a bed, people segment the actions into discrete steps and substeps by accomplishment of goals and subgoals, not by time per se (e.g., Tversky, Zacks, & Hard, 2008). Animations typically fail to segment processes into their natural steps. Finally, showing is not explaining. Animations can show some changes, but in and of themselves do not explain the causal connections. In fact, animations accompanied by explanations can improve understanding when compared with animations without explanations (e.g., Mayer, 2005).

### The roles of gesture in expressing and understanding thought

An underused and understudied possibility for effectively explaining dynamic systems is to use gestures. Gestures are actions; they should be natural for conveying actions (e.g., Cartmill, Beilock, & Goldin-Meadow, 2012; Hostetter & Alibali, 2008). Numerous studies have shown that people spontaneously gesture when explaining to themselves or to others (e.g., Alibali, Bassok, Solomon, Syc, & Goldin-Meadow, 1999; Alibali, Spencer, Knox, & Kita, 2011; Atit, Gagnier, & Shipley, 2014; Cartmill et al., 2012; Chu & Kita, 2011; Emmorey, Tversky, & Taylor, 2000; Ehrlich, Levine, & Goldin-Meadow, 2006; Engle, 1998; Goldin-Meadow & Beilock, 2010; Goldin-Meadow & Alibali, 1999; Goldin-Meadow, Kim, & Singer, 1999; Goldin-Meadow, Nusbaum, Kelly, & Wagner, 2001; Gukson, Goldin-Meadow, Newcombe, & Shipley, 2013; Hostetter & Alibali, 2008; Kang, Tversky, & Black, 2014; Schwartz & Black, 1996). In many cases, gestures carry information that is not carried in speech. Considerable research has shown that information carried solely by gesture can facilitate learning, thinking and understanding in both children and adults in a broad range of tasks including conservation (e.g., Church, Ayman-Nolley, & Mahootian, 2004; Ping & Goldin-Meadow, 2008), word learning (McGregor, Rohlfing, Bean, & Marschner, 2009), problem solving (Beilock & Goldin-Meadow, 2010; Singer & Goldin-Meadow, 2005; Tversky & Kessell, 2014), sentence memory (Thompson, Driscoll, & Markson, 1998), asymmetry (Valenzeno, Alibali, & Klatzky, 2003), math (e.g., Alibali & DiRusso, 1999; Cook, Duffy, & Fenn, 2013; Cook & Goldin-Meadow, 2006; Goldin-Meadow et al., 1999; Segal, Tversky, & Black, 2014), math analogies (Richland & McDonough, 2010), cyclical and simultaneous time (Jamalian & Tversky, 2012), story understanding (Beattie & Shovelton, 1999), and more.

### Gestures can represent and resemble action

Gestures are frequently produced spontaneously to express both structure and action (e.g., Atit et al., 2014; Cartmill et al., 2012; Chu & Kita, 2011; Emmorey et al., 2000; Enfield, 2003; Engle, 1998; Goldin-Meadow & Beilock, 2010; Gukson et al., 2013; Kang et al., 2014). In previous research showing effects of communicative gestures that convey actions, the gestures used have been single actions on visible objects, such as lifting a disk (Goldin-Meadow & Beilock, 2010), counting (Carlson, Avraamides, Cary, & Strasberg, 2007) or rotating an imagined object (Alibali et al., 2011; Chu & Kita, 2011; Schwartz & Black, 1996). The present research examines the role of an integrated sequence of gestures representing a sequence of actions on named rather than instantiated objects. In order to convey structure, action, or other concepts, gestures must be custom-crafted to represent the specific content. Like effective graphics, effective gestures should be congruent with the meanings they express. As for graphics, gestures can map meanings more directly than language. A sequence of pointing gestures in gesture space can map the relative spatial locations of landmarks in an environment, much like a schematic map (Emmorey et al., 2000). A circling gesture is a more direct and congruent representation of circling motion than the word “circling.” Gestures are themselves actions and can be three-dimensional so can represent complex manners of action more directly certainly than words and in many cases also more directly than flat diagrams or animations. Note that in these congruent mappings of meaning, the gestures both *represent* the concept to be conveyed and *resemble* the concept to be conveyed. Both the word “circling” and a circular motion of the finger represent circling motion but only the circular motion resembles circling. A circling gesture can be more readily apprehended than a word, which is an arbitrary mapping of meaning to sound requiring knowledge of the language.

### Neuroscience and action

Gesture, then, should have a special role in representing action for explanations and understanding. Gestures are spontaneously used to convey action and gestures can both represent and resemble actions. Neuroscience research also shows connections between thought, action and gesture. Watching actions performed by others, especially well-known actions, has been shown to activate regions of the brain involved in planning or making actions, a phenomenon known as *motor resonance* (e.g., Decety et al., 1997; Iacoboni, Rizzolatti & Craighero, 2004; Iacoboni et al., 1999; Molenberghs, Cunnington, & Mattingly, 2012; Rizzolatti & Craighero, 2004; Rizzolatti, Fogasse, & Gallese, 2001; Utihol, van Rooij, Bekkering, & Haselager, 2011). The general view is that this kind of motor mirroring serves action understanding. Seeing action gestures, then, should induce motor resonance, adding a layer of meaning and understanding of action.

This analysis suggests that gestures showing a sequence of actions could deepen understanding of the actions of a dynamic system, the goal of the present study. After considering previous research and extensive pretesting, we selected the four-stroke engine typically found in automobiles as a test platform. Previous research has used mechanical systems such as a bicycle pump, a pulley system or car brake, or biological systems such as the heart (e.g., Mayer, 2005). However, these systems do not have many differentiated actions or are already familiar to many undergraduates. An engine has several different kinds of integrated actions and is more complex and less known than the systems typically studied. Yet, it does not assume the background knowledge required in studies of chemistry, biology or physics. In the present study, students viewed one of two videos explaining the behavior of an engine accompanied by one of two types of gesture. The text of the explanation was exactly the same for both conditions and both videos were accompanied by the same rudimentary diagram of the engine showing the named parts in the proper configuration. In the action-gesture video, the explanation was accompanied by gestures that portrayed the actions of each part of the system, for example, opening, closing, expelling, exploding, igniting, compressing, reducing, letting in, rotating, descending, going in, going up, and going out. In the structure-gesture video, the explanation was accompanied by an identical number of gestures that portrayed the structure of each part of the system, for example, the crankshaft, the cylinder, the intake valve, the piston, the spark plug and the exhaust valve. In pretesting, two viewings of the video resulted in only chance performance on the knowledge test but four viewings led to a reasonable level of comprehension, above chance but not perfect, similar to previous work on learning complex environments (e.g., Taylor & Tversky, 1992).

Understanding was evaluated in several ways: by questions about structure and action that could be answered solely from the text, by student-created visual explanations and by student-created oral explanations to peers. We were especially interested in the students’ creations, their visual explanations and oral explanations because these require both understanding the information and reformulating it. If seeing action gestures creates a deeper understanding of action, those who viewed them should represent more action in their diagrams and include more action information in their verbal explanations by using more action words and more action gestures. Because structure is typically easier to learn than action and because both groups viewed a rudimentary diagram of structure, little or no benefit was expected from seeing the structure gestures.

## Method

### Participants

Fifty-nine (15 male) university students ranging in age from 20 to 36 years with an average age of 26 (*SD* = 3.50) participated in the study. All were native English speakers with no technology or engineering background and none had prior knowledge of the system to be learned. The study was approved by the Institutional Review Board (IRB) and all participants signed the stipulated informed agreement.

## Materials

**Fig. 1:**
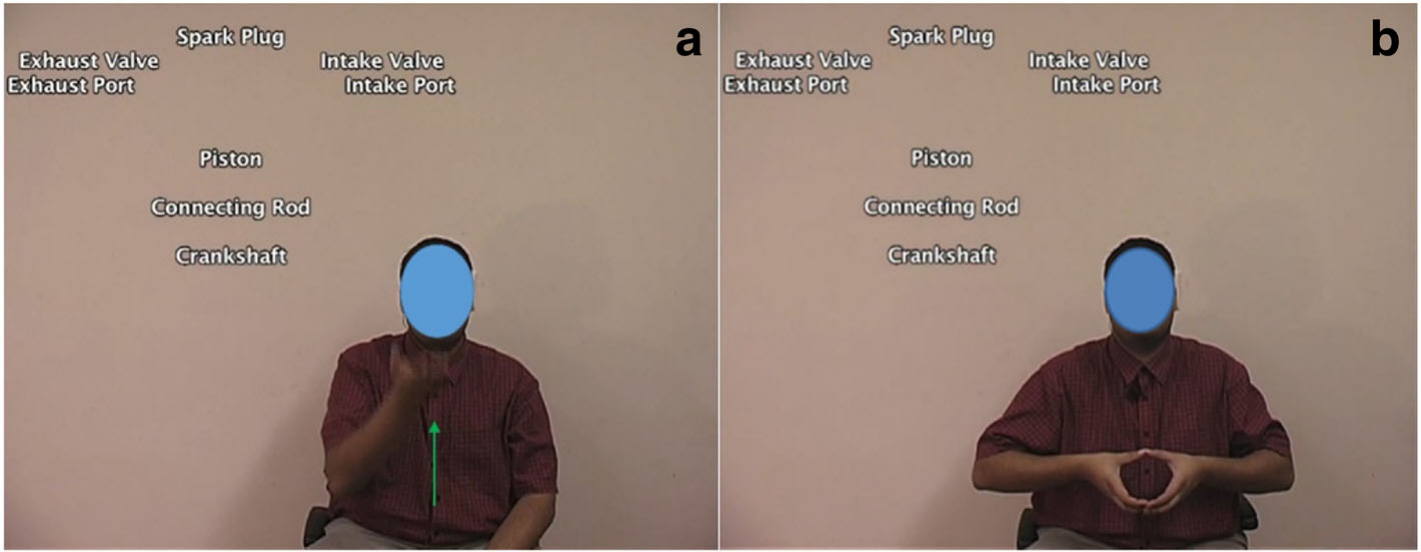
Still shots from the action (**a**) and structure (**b**) videos showing the superimposed diagrams. The speaker’s head was not blurred for participants

We created two videos explaining how a four-stroke engine works with the help of a professional video producer. The videos were identical in language and number of gestures but differed in kinds of gestures. A rudimentary diagram showing the labels of the parts in their proper configuration was superimposed in front and to the side of the speaker. The explanations began with an introduction overviewing the structure using deictic gestures that pointed to the named parts. The core portion of the explanation was a step-by-step explanation of the processes comprising the workings of the system. The final portion of the explanation explained how the process caused the car’s wheels to rotate. Because the diagram showing the structure was always in view and because the introduction to both explanations overviewed the system structure, the gestures exemplifying structure served primarily as a control and were not expected to affect performance on the questions. For the core portion of the explanation, in the action video the speaker used gestures that portrayed the action of each part. In the structure video the speaker used only gestures that pointed to the location of the parts of the system and showed the shape of each part as the process was explained. The accompanying verbal script (Appendix 1) explained both the locations of the parts and the actions of the parts identically. Figure 1 shows snapshots of the two instructional videos.

The information in the script was categorized as structure or action, and gestures appropriate for each were devised. For the action gesture video, the explainer showed the rotational motion of the crankshaft, the direction of the piston’s movement, the flow of fuel and air, the movement of the intake and exhaust valves, and so on with his hands. The action gestures were timed with the verbs describing the actions. The action gestures corresponded spatially with the approximate locations of the corresponding parts so that the action gestures naturally included some rudimentary information about overall structure.

For the structure gesture video, the explainer used his hand(s) to show the successive shapes of the crankshaft, piston and cylinder, and showed the positions of the piston, crankshaft, spark plug, intake port, intake value, exhaust port, exhaust valve, and mixture of fuel and air. The structure gestures were timed with the names of the described parts and located appropriately.

To eliminate any biasing effects of lexical stress (van Heuven, 1988; Field, 2005), the explainer practiced the script several times, making sure to stress the actions and the parts for both videos. The action gesture video took 3 min 29 s and the structure gesture video took 3 min 32 s.

### Knowledge assessments

The verbal knowledge test (Appendix 2) was based solely on the information in the script. It had 20 recognition questions, 16 True/False, and 4 multiple-choice questions, each worth 1 point. Of the 16 True/False questions, 8 queried action and 8 queried structure. Action questions referred to movement, or causal relations of the parts and their consequences. Structure questions referred to shapes and positions of the parts of the system. Four multiple-choice questions queried general knowledge. The questions were presented in random order. A second assessment was a diagramming task. Participants were asked to create a visual explanation of how a four-stroke engine works based on what they learned from the video. Finally, participants made a video to explain the workings of the four-stroke engine to a peer.

### Procedure

The participants were seated at a table in front of a laptop computer with a 15.4-inch screen. They were randomly assigned to either the action gesture or the structure gesture condition. The participants were then told: “Today, your job is to watch a video of how a four-stroke engine works four times in a row and explain the concept in the video to a peer coming later. However, since you are not directly explaining a concept, your explanation will be videotaped and showed later either to him or her. He or she will learn about the concept from your explanation.” The participants were not allowed to take notes or to pause or stop the video. The experimenter left the room while the participants watched the video. After watching the video four times, the participants were given the verbal knowledge test and the diagramming task, and then made a video explaining the system to a peer. The video camera was set opposite the participant 3 m away. Participants were allowed to spend as much time as they wanted carrying out this task.

### Gesture coding

Gestures that conveyed relevant semantic content were coded and analyzed. A gesture was defined as a movement of hand(s) accompanied by speech to express an idea or meaning. A gesture unit was defined as “the period of time between successive rests of the limbs (McNeill, 1992).” Movement of the hand(s) starting from a resting position and returning to a resting position was regarded as one gesture. If the hands did not return to a resting position between two gestures, the boundary was defined by a pause in motion and an obvious change in shape or trajectory. When a participant used both hands simultaneously to describe one object, concept, or part, it was regarded as one gesture. If a participant used both hands and one described an object, a concept, or a part and the other hand a different concept, the gestures were coded as two different gestures. Beats, which serve to advance the discourse, and emblems, which have conventionalized meanings like “OK,” were excluded as were a very small number of metaphoric gestures expressing abstract ideas.

Participants’ gestures were categorized in two ways. Gestures were coded as imitated when the hand shapes were the same as the viewed gestures or invented when the hand shapes differed. The semantic content of the gestures was coded as action or structure depending on the properties of the engine they exemplified. Action gestures showed the action of a part or a process of a system. They were frequently schematic, showing only the direction of the action. Structure gestures showed the position or structure of a part, for example, showing the contour of a part or pointing to relative position of a part. Blends, where a gesture carried both action and structure information, were coded as an action gesture. Blends were always invented gestures.

Interrater reliability was assessed on a randomly selected set of 240 gestures (18 %) by a second coder who was trained and blind to the experimental design. Cohen’s kappa agreement for categorizing gestures was 0.97 (*p* < .01) for action and structure gesture, and 0.66 (*p* < .01) for categorizing gestures into invented and imitated. Discrepancies were resolved by discussion.

### Speech coding

Participants’ verbal descriptions were segmented into propositions (i.e., the unit of meaning in a sentence). The information units were coded as *action*, *structure* or *other*. Most propositions with “is-a” or “has-a” were coded as *structure*. For example, “…on each side there are two valves…” was coded as one *structure* information unit. Propositions containing action verbs, either by or on a part, were coded as *action*. Additionally, propositions with “has-a” or “is-a” were coded as action if the argument was about action, for example, “…that’s one half cycle rotation…” was coded as action but “…there is the compression phase…” was coded as structure. Although *compression* is an action, *phase* is of structure.

*Other* information included greetings, such as “Good evening,” introductory information such as “I’m going to explain how a four-stroke engine works,” and meta-comments such as “…let me tell you a little bit more about each stage….”

Interrater reliability was assessed on a randomly selected set of 570 speech information units (22 %) by a second coder who was trained and blind to the experimental design. Cohen’s kappa agreement for categorizing information units was 0.78 (*p* < .01).

## Results

### Knowledge test

**Fig. 2:**
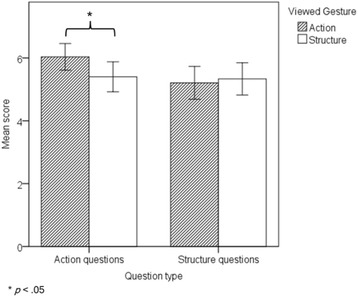
Mean scores in the knowledge test. Error bars represent standard errors of the means

An item analysis of the knowledge test revealed that one of the eight action questions and one of the eight structure questions had low reliability with the remaining questions and were not pure action or structure, but relied on knowledge of both. The action question was: “A byproduct of air and fuel is pushed by a piston and goes out through an exhaust port,” and the structure question was: “The piston is located closer to the crankshaft in the combustion phase than in the exhaust phase.” Those items were deleted so that performance was analyzed for seven action questions and seven structure questions; the means appear in Fig. 2. Overall performance was good. The action group (mean (*M*) = 6.03, *SD* = 1.12) performed better on the action questions than the structure group (*M* = 5.40, *SD* = 1.28), *F*(1,57) = 4.12, mean squared error (MSE) = 1.44, *p* < .05, ${\eta}_p^2=.07$. There were no group differences on the structure questions between the action group (*M* = 5.21, *SD* = 1.37) and the structure group (*M* = 5.33, *SD* = 1.37) (*p* = .73). Likewise, there was no group difference between the action group (*M* = 13.93, *SD* = 2.28) and the structure group (*M* = 13.33, *SD* = 2.80) in total scores that included the general knowledge questions in addition to the questions about structure and action (*p* = .37) and no interaction between group and question type, *F*(1,114) = 2.57, *p* = .11.

### Visual explanations

**Fig. 3:**
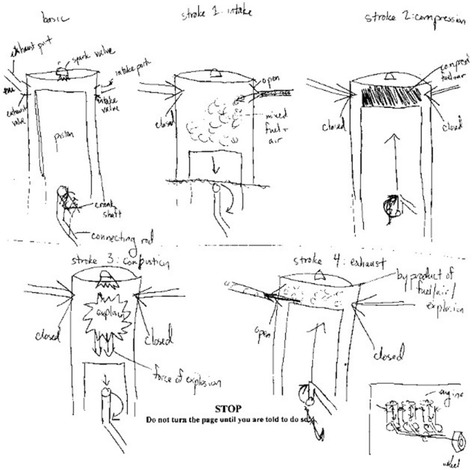
Visual explanation of a participant who saw action gestures

**Fig. 4:**
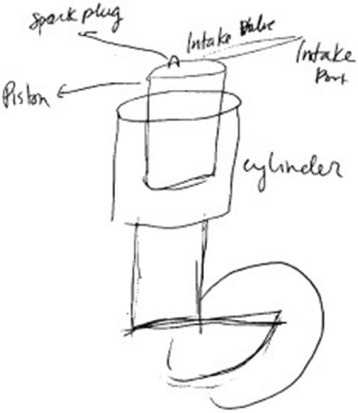
Visual explanation of a participant who saw structure gestures

Examples of diagrams that participants produced in creating visual explanations are shown in Figs. 3 and 4. The diagrams were analyzed for inclusion of four key visual components that reflected action and structure information. The experimenter and another coder blind to conditions coded all the diagrams and resolved any differences by discussion. The components and reliability were as follows: action arrow, kappa = 0.63 (*p* < .01); action effect, kappa = 0.65 (*p* < .01); labeling arrow, kappa = 0.60 (*p* < .01); labeling line, kappa = 0.73 (*p* < .01). Action arrows showed movement, for example, of a part or the flow of a mixture of air and fuel. Action effects were depictions of actions, such as ignition, explosion or compression as in the bubbles and jagged circle in Fig. 3. Labeling arrows or lines connected names with the corresponding depiction of a part as in Fig. 4.

**Fig. 5:**
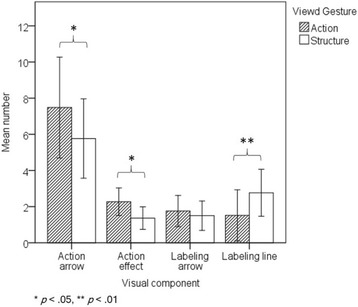
Mean number of visual component types produced in visual explanations by viewed gestures. Error bars represent standard errors of the means

Poisson regression analyses were used to model count variables under the assumption that the conditional means equal the conditional variances. The means of the diagram components by gesture condition appear in Fig. 5. Those who watched action gestures produced more visual components (*M* = 18.38, *SD* = 11.36) than those who viewed structure gestures (*M* = 15.77, *SD* = 10.87), (*χ* ^2^(1, *N* = 59) = 7.26, *p* < .05). They also produced more action arrows (*M* = 7.48, *SD* = 7.51) than the structure group (*M* = 5.77, *SD* = 6.02), (*χ* ^2^(1, *N* = 59) = 6.90, *p* < .05) as well as more action effects (*M* = 2.28, *SD* = 2.05) than the structure group (*M* = 1.37, *SD* = 1.71), (*χ* ^2^(1, *N* = 59) = 1.99, *p* < .05). By contrast, the structure group produced more labeling lines (*M* = 2.77, *SD* = 3.56) than the action group (*M* = 1.52, *SD* = 3.81), (*χ* ^2^ (1, *N* = 59) = 7.04, *p* < .01). No differences were observed in the number of labeling arrows (*p* = .44). Thus, effects of the viewed gestures were apparent in the diagrams. Those who saw action gestures showed far more action in their diagrams in the form of arrows showing direction of movement and depictions of actions. Conversely, those who saw structure gestures used more lines to label parts.

### Completeness of visual explanations

Participants’ visual explanations were analyzed for completeness. Diagrams were coded as *complete* if they included all four steps of the process and *incomplete* otherwise. The names of the steps alone did not count as complete. Those who had seen action gestures produced more complete diagrams (25 out of 29) that included all four steps than those who saw structure gestures (19 out of 30), *χ* ^2^(1, *N* = 59) = 4.07, *p* < .05.

### Videoed explanations

**Fig. 6:**
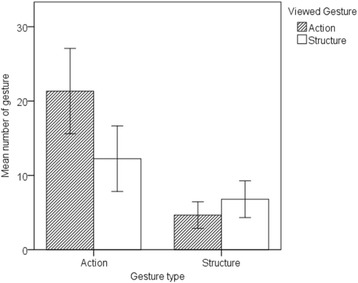
Mean number of action and structure gestures produced by viewed gesture. Error bars represent standard errors of the means

The participants’ videoed explanations were analyzed for gesture and language. One video was not recorded due to equipment malfunction. Two participants from the action group and three from the structure group never used their hands but were included in the analyses because not producing gestures is a behavioral pattern, if an infrequent one. The average explanation time was 177.14 s (*SD* = 56.84) for the action group and 152.34 s (*SD* = 55.94) for the structure group, a difference that did not reach significance (*p* = .10). Even though they had seen the same number of gestures, those who had viewed action gestures produced more gestures (*M* = 26.55, *SD* = 19.09) than those who had viewed structure gestures (*M* = 20.00, *SD* = 16.09), (*χ* ^*2*^(1, *N* = 58) = 13.34, *p* < .01). Figure 6 shows the mean numbers of gestures of each type by viewed gesture.

**Fig. 7:**
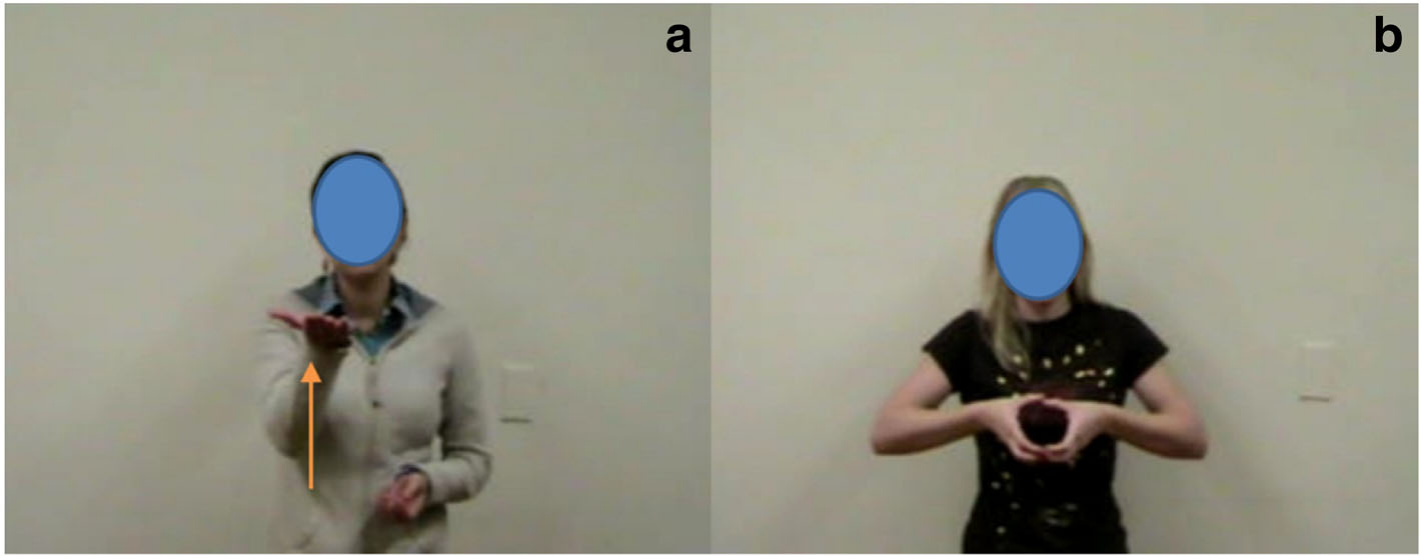
Examples of gestures produced in videoed explanations. The left panel shows a participant making an action gesture; the right panel shows a participant making a structure gesture

Produced gestures were coded as action or structure. An example of each appears in Fig. 7.

Irrespective of viewing condition, participants produced more action gestures than structure gestures. Participants who had viewed action gestures produced an average of 21.62 (*SD* = 15.21) action gestures and an average of 4.93 (*SD* = 5.28) structure gestures. Those who had viewed structure gestures produced an average of 12.90 (*SD* = 11.79) action gestures and an average of 7.10 (*SD* = 6.67) structure gestures. A split-plot analysis of variance (ANOVA) analysis revealed that there was an interaction between viewed gesture and produced gesture, *F*(1,56) = 13.16, *MSE* = 65.44, *p* < .01, ${\eta}_p^2=.19$.

Although the differences in explanation time were not significant, explanations by participants who had viewed action gestures were on average longer. Therefore, the analyses were repeated on gesture rate. The same findings emerged. Those who had viewed action gestures produced 7.00 (*SD* = 4.15) action gestures per minute and 1.51 (*SD* = 1.41) structure gestures per minute. Those who had viewed structure gestures produced of 4.87 (*SD* = 3.55) action gestures per minute and 2.62 (*SD* = 2.41) structure gestures per minute. A split-plot ANOVA analysis revealed an interaction between viewed and produced gestures; those who had viewed action gestures produced action gestures at a higher rate and those who had viewed structure gestures had produced structure gestures at a higher rate, *F*(1,56) = 11.13, *MSE* = 6.81, *p* < .01, ${\eta}_p^2=.17$.

Combining both groups, we found that gesture use correlated with number of visual components in the diagrams and with scores on the knowledge test, evidence that better understanding is also expressed visually, in gestures and diagrams. The number of action gestures correlated with number of action arrows (*r* = .280, *p* < .05) and with number of action effects (*r* = .282, *p* < .05) in the visual explanations. Knowledge test scores were marginally correlated with frequency of action gestures (*r* = .234, *p* = .078), but not with frequency of structure gesture (*r* = −.036, *p* = .791).

### Invented and imitated gestures

**Fig. 8:**
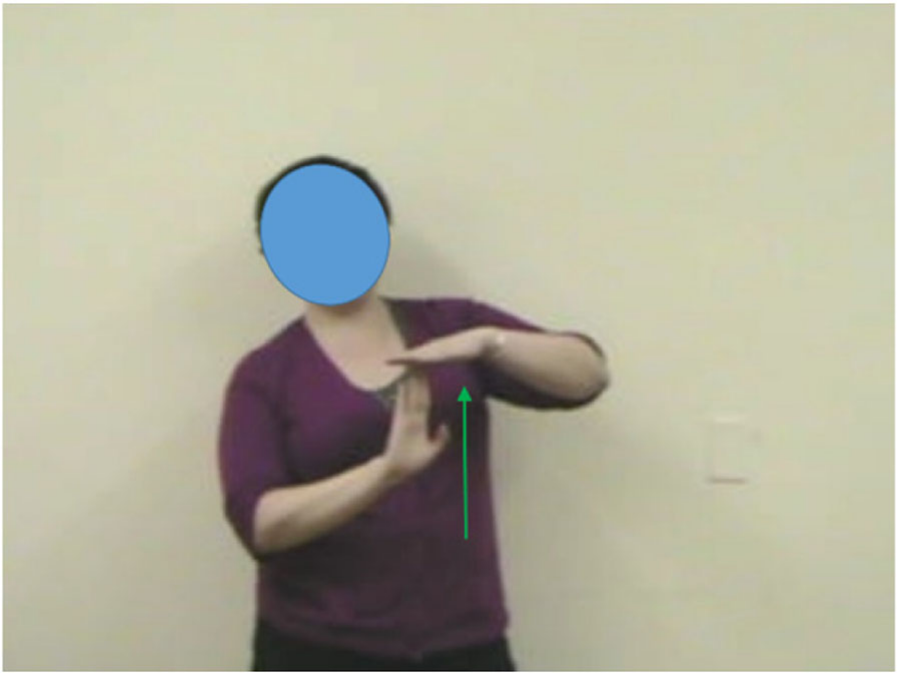
An invented gesture demonstrating a piston moving up (cf. Figs. 1a and 7a)

Most of the gestures participants produced were inventions, not imitations of what they had seen. In communicative situations, gesture mimicry is common (e.g., Holler & Wilkin, 2011; Mol, Krahmer, Maes, & Swerts, 2012). Thus, invented gestures are especially indicative of deep understanding because they are creations of the individuals from their own understanding rather than copies of what they viewed. Any structural gestures produced by those who viewed the action gesture video and any action gestures produced by those who viewed the structure gesture video were a-priori invented. Also, any additional action gestures produced by those who viewed action gestures, or additional structural gestures produced by those who viewed structure gestures, were coded as invented. Finally, gestures that used different hand shapes from those that had been viewed were coded as invented. For example, the speaker in panel (a) of Fig. 7 described a piston moving up. This gesture was coded as imitated because in the action instructional video the speaker spread his right hand with the palm up and moved it upward in the same way. In contrast, the participant in Fig. 8 represented the same action of the piston but with a different hand shape; in addition she portrayed a piston with her left hand and a rod with her right hand which connects the crankshaft and a piston, pushing the piston up.

**Fig. 9:**
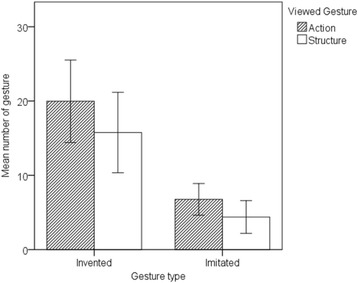
Average invented and imitated gestures by gesture type and viewed gesture. Error bars represent standard errors of the means

Gestures similar in hand shape to those viewed were coded as imitated. The frequencies of invented and imitated gestures are shown in Fig. 9. Those who saw action gestures produced 19.97 (*SD* = 14.96) invented gestures and 6.76 (*SD* = 5.74) imitated gestures on average. Those who had viewed structure gestures produced 15.76 (*SD* = 14.58) invented gestures and 4.38 (*SD* = 5.93) imitated gestures on average.

No interaction was found between gesture viewed and gesture produced (*p* = .62). Participants produced more invented than imitated gestures, *χ* ^*2*^(1, *N* = 58) = 9.26, *p* < .01. Those who had viewed action gestures produced both more invented (*χ* ^*2*^(1, *N* = 58) = 12.35, *p* < .01) and more imitated gesture (*χ* ^*2*^(1, *N* = 58) = 6.45, *p* < .01) than those who had viewed structure gestures.

Analyses of gesture rate corroborated most of these findings. Those who had viewed action gestures produced an average of 6.43 (*SD* = 4.18) invented gestures per minute and an average of 2.14 (*SD* = 1.74) imitated gestures per minute. Those who had viewed structure gestures produced an average of 5.80 (*SD* = 4.10) invented gestures per minute and an average of 1.76 (*SD* = 2.52) imitated gestures per minute. There were no differences in gesture rate by viewed gesture. Across conditions, participants produced more invented gestures than imitated gestures, *t*(57) = 7.26, *p* < .01, *d* = 1.27. There was no interaction between viewed and produced gesture types (*p* = .83) nor did two groups differ in invented gesture per minute (*p* = .57) and imitated gesture per minute (*p* = .51).

### Speech analysis

**Fig. 10:**
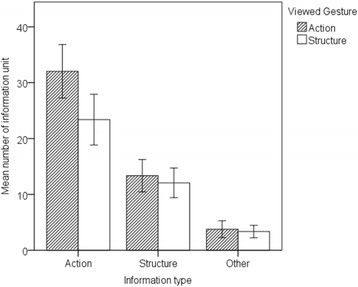
Mean number of information units by information type in the two groups. Error bars represent standard errors of the means

Supporting the claims that action information is both more important and harder to convey, of the total of 2550 information units in the speech corpus, 1607 conveyed *action*, 737 conveyed *structure*, and 206 conveyed *other* information. Those who had viewed action gestures produced a total of 1425 information units, 929 conveying action, 387 conveying structure, and 109 conveying other information. Those who had viewed structure gestures produced a total of 1125 information units, 678 conveyed action, 350 conveyed structure, and 97 conveyed other information. Figure 10 shows the mean types of information produced by those who had viewed action and structure gestures. Poisson regression analysis revealed an interaction between gesture viewed and type of speech, *χ* ^*2*^(2, *N* = 58) = 6.55, *p* < .05. Those who had viewed action conveyed relatively more action information in their speech than those who viewed structure gestures. That interaction held when *other* information was excluded from the analysis, *χ* ^*2*^ (2, *N* = 58) = 5.76, *p* < .05. The frequency of producing the various information types differed, *χ* ^*2*^ (2, *N* = 58) = 905.11, *p* < .01. Overall, participants spoke more about action than about structure (*p* < .01), and more about structure than other (*p* < .01).

### Proportion of information type in speech

**Fig. 11:**
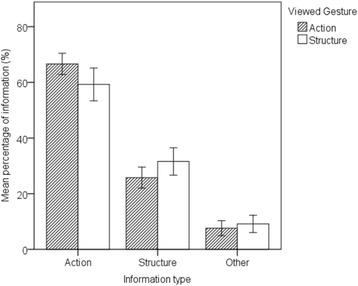
Mean percentage of information units by the two groups. Error bars represent standard errors of the means

Because those who had viewed action gestures produced more speech, the proportions of action, structure, and other information units were analyzed by viewed gesture; the means appear in Fig. 11.

A univariate ANOVA corroborated a higher percent of action information, *F*(2, 168) = 348.23, *p* < .01, ${\eta}_p^2=.81$. Post-hoc (Tukey’s HSD) confirmed this; more *action* information was conveyed than *structure* and other information units (*p* < .01), and more *structure* information was conveyed than *other* information units (*p* < .01). A split-plot ANOVA was administered and showed that viewed gesture and speech information interacted, *F*(2, 112) = 3.44, *MS* = 190.11, *p* < .05, ${\eta}_p^2=.06$. Those who had viewed action gestures spoke relatively more about action than those who had viewed structure gestures; similarly, those who had viewed structure gestures spoke relatively more about structure. When *other* information was excluded, the same interaction held between viewed gesture and type of information produced, *F*(1, 56) = 4.37, *MSE* = 288.10, *p* < .05, ${\eta}_p^2=.07$.

## Discussion

Dynamic systems are pervasive in our lives, but are often difficult to understand. Dynamic systems typically have a structural layer, the parts and their interrelations, as well as a dynamic layer, the actions, changes, behaviors, processes, consequences and causes that occur over time. The structural layer is normally easier to convey and easier to comprehend than the dynamic layer. The structural layer is static but the dynamic layer can include many different kinds of actions and contingencies or consequences. Here, we asked whether accompanying explanations of dynamic systems with a sequence of gestures that represent the actions of the parts of the system could enhance understanding of the dynamics of the system. One group of students watched an explanation accompanied by gestures representing the actions of the parts; another group of students watched the same verbal explanation but accompanied by gestures showing the forms of the parts and their spatial array. The verbal explanation was the same for both explanations. Both types of gestures are common in spontaneous explanations. A schematic diagram of the spatial array of the names of the parts accompanied both explanations.

Viewing gestures representing action, as opposed to gestures conveying structure in explanations of dynamic systems, had profound effects on participants’ understanding of the dynamics of the system. The deeper understanding of the dynamics of the system was expressed in many ways; first, in better performance on questions about the action of the system, questions that could be answered solely from the shared verbal script. Even more revealing, the deeper understanding was expressed in students’ own creations, in their sketched visual explanations of the system and in their videoed explanations of the system. The deeper understanding was revealed in their diagrams, in their words, and in their own invented gestures. The visual explanations of those who had seen action gestures contained more arrows indicating direction of movement; they also contained more depictions of the various actions, such as explosion or ignition. These features were neither in the viewed diagram nor in the video; they derived from participants’ own understanding; they derived, if you will, from their mental models of the system.

Importantly, seeing action gestures provided participants with more complete and comprehensive understandings of the system. The visual explanations revealed that far more of those who had seen action gestures distinguished and included all four stages of the system than those who had viewed structure gestures.

In their own oral explanations, participants from both groups devoted three times as many words and three times as many gestures to explaining the dynamics of the system as to explaining the structure. This is dramatic evidence that the dynamics of a complex system require more explanation than the structure.

Deeper understanding of the system dynamics was evident in the oral explanations of the systems by participants who had seen action gestures. Their explanations contained more words expressing action, despite having heard the same words as those who had viewed gestures conveying structure. Both groups accompanied their explanations with many gestures, more for action than for structure. The majority of gestures produced by participants in both groups were inventions by the participants. The gestures produced had different forms (hand shapes) from those they had seen; that is, they were not close copies of viewed gestures. Those invented gestures can be regarded as synonyms rather than quotes and constitute evidence that they derived from participants’ deep understandings rather than from superficial memory of what they had seen.

Overall, the results demonstrate far-reaching effects of action gestures on understanding. Because the language was the same for both groups, gesture affects understanding over and beyond language. Watching an explanation of a dynamic system accompanied by gestures representing the sequence of actions led to deeper understanding of the dynamics of the system compared to seeing gestures representing the structure of the parts. The deeper understanding was reflected in a better grasp of the stages of the system, better performance on questions about the dynamics of the system, and more action information expressed in diagrams, words and invented gestures. Gestures conveying structure had little effect on understanding structure, nor were any effects expected. Structural information is easier to grasp than dynamic information, and a diagram showing structure was used in the viewed explanation.

Numerous studies have shown that people express information in gestures that they do not express in speech, important information about their thinking, including structure, action, and more (e.g., Bavelas, 1994; Church & Goldin-Meadow, 1986; Emmorey et al., 2000; Garber, Alibali, & Goldin-Meadow, 1998; Goldin-Meadow, 2003; Goldin-Meadow & Alibali, 1999; Heiser, Tversky, & Silverman, 2004; Jamalian, Giardino, & Tversky, 2013; Jamalian & Tversky, 2012; Kirsh, 2013). Integrated sequences of gestures can create virtual models of complex spaces or complex sequences of actions (Emmorey et al., 2000; Heiser et al., 2004; Kang et al., 2014; Kirsh, 2013; Tversky, Heiser, Lee, & Daniel, 2009; Jamalian et al., 2013). Here, we transferred gestures for expression to gestures for teaching and learning; we found that an integrated series of gestures congruent with action can deepen understanding of the actions of a dynamic system.

This study is by no means the first to demonstrate the power of gesture to instill knowledge. Examples abound, in math (e.g., Cook et al., 2013), word learning (McGregor et al., 2009), conservation (Ping & Goldin-Meadow, 2008), understanding symmetry (Valenzeno et al., 2003), understanding simultaneity (Jamalian & Tversky, 2012). However, this is the first demonstration of the efficacy of an orchestrated sequence of conceptually congruent gestures to instill deeper understanding of a dynamic system and to demonstrate that deeper understanding in students’ own words, gestures, and diagrams as well as in a test of knowledge. A further benefit of gesture is that it is “low-tech,” nothing more is needed than the tools that we carry with us at all times, our hands and our bodies.

Expressing knowledge visually by means of gesture bears similarities to expressing knowledge in graphics. Both gestures and graphics can abstract, segment, and integrate information to be conveyed or understood (e.g., Tversky & Kessell, 2014; Tversky, 2011; Tversky et al., 2009). Diagrams are typically multimodal, incorporating and integrating both marks in space, their sizes, formats, and places in space, and also words, symbols, and more to create complete messages. So, too, are gestures; they are typically an integral part of a complete multimodal message (e.g., Engle, 1998). In much diagrammatic communication—think of maps, science diagrams, assembly instructions—the visual-spatial features of meaning form the core of the message; the words and symbols annotate (e.g., Netz, 1999). There are parallel cases for gesture; that is, the sequence of gestures form the core of the communication, and the words serve to annotate the gestures (e.g., Emmorey et al., 2000; Kirsh, 2013). In many instances, the three—gesture, talk, and diagram—work together, complementing and supplementing each other (e.g., Engle, 1998; Kang et al., 2014; Heiser et al., 2004). Examples abound, for example, in sports, dance, musical instruments, cooking, and more. The present results along with the previous studies make a strong case for incorporating well-crafted gestures and other forms of visual communication in teaching, especially of dynamic systems that entail actions in time. Dynamic systems are typical of Science, Technology, Engineering and Math (STEM), but also other domains such as history.

In most, if not all, cases, the use of gesture to form the core of messages or to complement, disambiguate, and enrich words seems to be because the information is easier to express and more precise in gesture than it words. In other words, it is more direct and more natural to show than to tell. In addition, information about space and action is often far more precise in gesture than in words. Pointing to the exact position of a part of an object can be more precise that describing the position; showing the motion of an object can be more precise than describing the motion. As Talmy (1983, 1988, 2000) analyzed and others have documented (e.g., Daniel & Tversky, 2012; Denis, 1997; Tversky & Lee, 1998), words in languages all over the world schematize information about space and action in space into rough categories like “close” and “far,” “here” and “there,” “up” and “down,” “forward” and “backward,” “push” and “pull,” and “turn.” Deictic terms like “here” or “this way” or “like this” accompanied by a gesture save the many words that would be needed to adequately describe the place or the direction or the manner of the action. The same word, “lift,” is used whether an object is heavy or light, but the gestures change (Beilock & Goldin-Meadow, 2010). Do I *push* with a finger or a hand or a handle? With one hand or two? The word “rotate” does not specify the plane of rotation nor does it specify the hand position, the strength needed, or whether a tool is required; gestures can readily do all that. Thus, the spatial and action information conveyed in gesture disambiguates and clarifies information that may be ambiguous or imprecise in speech, yielding greater accuracy in communication (e.g. Heiser et al., 2004). Just as gestures are effective in communicating information more precisely and directly to others, they are also more effective than words alone in comprehending and communicating information for self (e.g., Jamalian et al., 2013).

Many have analyzed the close connections between gesture and action, calling attention to phenomena like motor resonance (e.g., Rizzolatti & Craighero, 2004) and postulating mediation through them (e.g., Cartmill et al., 2012; Goldin-Meadow & Beilock, 2010; Hostetter & Alibali, 2008; Holler & Beattie, 2003; Kirsh, 2013; Kita & Özyürek, 2003; Tversky & Kessell, 2014). Building on those insightful analyses, we propose a more direct relationship between gestures and representations of space and action. Concepts of space and action (and much more) map naturally and directly to places and actions of the hands and the body (Cartmill et al., 2012; Tversky, 2011, 2015; Tversky & Kessell, 2014; Tversky et al., 2009). The hands and the body both are in places and act in space and, therefore, can readily represent places and actions in space. This natural mapping as well as the increased precision of gesture over words makes gestures ideal for representing space and action both for self and for others. Gestures express meanings directly and, in some cases, can prime the relevant words (e.g., Krauss, 1998). Gestures are primary to meaning, not secondary.

## Conclusion

Before there were words, there were gestures, both ontogenetically and phylogenetically (e.g., Call & Tomasello, 2007). Babies typically gesture before they speak (e.g., Iverson & Goldin-Meadow, 2005). In an analysis of the evolution of language drawing on the neurological basis of mirror neurons, Rizzolatti and Arbib (1998) postulate that gestures, especially action gestures, grew out of abbreviated actions. Given that the same neurons in the premotor cortex in monkeys fire when monkeys perform hand actions and view hand actions, abbreviated hand actions could be used to communicate and understand intentions to perform specific actions. They further note that the brain substrate for mirror neurons in monkeys is homologous with Broca’s area in humans, a region long known to be involved in language production and understanding in humans. More recent research in neuroscience implicates Broca’s area in action understanding as well (e.g., Fadiga & Craighero, 2006; Fadiga et al., 2006). Communication canonically began face-to-face in small groups. Face-to-face communication occurs in specific contexts, often around a task or topic related to the context. Aspects of context can be and are used in conversations, pointed to, manipulated, and often given new meanings (e.g., Clark, 1996). As such, face-to-face communication could and can rely on gestures and props, using gestures to bring props in the context into the conversation. In fact, our vocabularies for certain domains are sparse, crude, abstract and ambiguous, even for concrete domains central to our existence, faces, space, and action. Gestures can be more precise and show more nuances than words. Gestures are actions in space, and thereby provide a natural and direct mapping for representing space and action. Gestures are powerful tools for thinking and communicating because they both represent and resemble.

## Appendix 1: Script for the instructional video

Today, I am going to explain how a four-stroke engine works. Almost all cars currently use what is called a four-stroke engine to convert gasoline into motion. A four-stroke engine to the cycle refers to a series of processes including intake, compression, combustion and exhaust. The series of processes happen inside the cylinder, which includes the intake valve, the intake port, the spark plug, the exhaust valve, the exhaust port, the piston, the connecting rod and the crankshaft.

Each cycle entails two rotations of the crankshaft for engines fueled by diesel or gasoline. Understanding the cycle’s four strokes is a key to understanding how the engine type works. “Four-strokes” to the cycle takes place continuously as the engine runs. In practice, this cycle happens one after the next in every cylinder of the engine.

### Intake

The beginning of the cycle starts at what is known as “top dead center.” The first half rotation of the crankshaft pulls the piston downward inside the cylinder, reducing pressure inside. As the piston descends, the intake valve is pulled open, letting in a mixture of forced fuel and air.

### Compression

The second half rotation of the crankshaft pushes the piston back up again inside the cylinder, compressing the fuel and air mixture as the intake valve closes.

### Combustion

The third half rotation of the crankshaft is known as the combustion stroke. At the end of the compression stroke, a spark plug ignites the combustible mixture of fuel and air. This small explosion pushes the piston downward again in the cylinder through its power stroke.

### Exhaust

The final stroke is known as the exhaust stroke. After the power stroke, the last half rotation of the crankshaft pushes the piston upwards in the cylinder for a second time, expelling the byproduct of the fuel and air combustion. As the crankshaft pushes the piston up, the exhaust valve opens, allowing the byproduct to go out.

In this process, the linear motion of the piston is converted into rotational motion by the crankshaft and this rotational motion is then used to rotate the car’s wheels.

## Appendix 2: Knowledge test

*Please choose a correct answer, or mark each of these statements are true (T) or false (F).

When the intake valve is pulled open, air and fuel move inside the cylinder. ( )

The crankshaft is attached to the wall of the cylinder. ( )

The piston is above the cylinder. ( )

The byproduct enters the cylinder when the crankshaft pushes the piston downward. ( )

The exhaust valve is located between the spark plug and the intake valve. ( )

In the exhaust phase, the piston moves upwards by rotation of the crankshaft. ( )

In the compression phase the piston is located closer to the intake valve than in the intake phase. ( )

From where does a mixture of air and fuel enter the cylinder? ( )

(1) intake valve (2) intake port (3) cylinder (4) exhaust valve

In the combustion phase, the mixture of fuel and air expands when the piston is pushed up within the cylinder. ( )

How many spark plugs does the cylinder have in one cycle? ( )

(1) one (2) two (3) three (4) four

The piston is located closer to the crankshaft m the combustion phase than in the exhaust phase. ( )

How many times does the crankshaft rotate in each cycle? ( )

(1) one (2) two (3) three (4) four

The piston is attached to the wall of the cylinder. ( )

A byproduct of air and fuel is pushed by a piston and goes out through an exhaust port. ( )

The linear motion of the crankshaft makes the car’s wheels rotate. ( )

A mixture of air and fuel is located above the piston. ( )

In the intake phase, the rotational motion of the crankshaft pulls the piston downward. ( )

The piston is below the crankshaft. ( )

In which stage does the engine get most of its power? ( )

(1) intake (2) combustion (3) compression (4) exhaust

In the compression phase, the piston is pushed back up again inside the cylinder. ( )

STOP

Do not turn the page until you are told to do so!
